# Gender effects and sexual-orientation impact on androstadienone-evoked behavior and neural processing

**DOI:** 10.3389/fnins.2014.00195

**Published:** 2014-07-31

**Authors:** Jacqueline Krajnik, Kathrin Kollndorfer, Karl-Heinz Nenning, Johan N. Lundström, Veronika Schöpf

**Affiliations:** ^1^Department of Biomedical Imaging and Image-Guided Therapy, Medical University of ViennaVienna, Austria; ^2^Department of Biomedical Imaging and Image-Guided Therapy, Computational Image Analysis and Radiology Lab, Medical University of ViennaVienna, Austria; ^3^Department of Clinical Neuroscience, Karolinska InstitutetStockholm, Sweden; ^4^Monell Chemical Senses CenterPhiladelphia, PA, USA; ^5^Department of Psychology, University of PennsylvaniaPhiladelphia, PA, USA

**Keywords:** androstadienone, chemosignals, gender-effect, sexual-orientation, neuronal processing, psychophysiological status, behavior

## Abstract

In humans, the most established and investigated substance acting as a chemosignal, i.e., a substance that is excreted from the body, is 4,16-androstadien-3-one (AND). AND, which is found in sweat and saliva, is known to be responsible for influencing several variables, such as psychophysiological status, behavior, as well as cortical processing. The aim of the present review is to give insight into the variety of AND effects, with special regard to specific cross-sexual characteristics of this putative human chemosignal, emphasizing the neural activation patterns and factors such as contextual conditions. This review highlights the importance of including those contributing factors into the analysis of behavioral as well as brain-related studies.

## Introduction

There is evidence for a sexual dimorphism of the brain (e.g., Coffey et al., 1998; Sacher et al., 2013) and new medical theories point out the importance of gender specific medicine in terms of risk-factors, epidemiology and treatment outcomes (e.g., Mielke et al., 2014). In order to extract essential and comprehensive information from behavioral as well as functional imaging studies, including gender, as an essential factor is of great interest (Sacher et al., 2013). This review highlights this importance for chemosensory science in which chemosignals are known to evoke a gender specific effect themselves.

The term “pheromones” (so-called chemosignals) was coined in 1959 by Peter Karlson and Martin Lüscher, who defined them as “substances which are secreted to the outside by an individual and received by a second individual of the same species, in which they release a specific reaction, for example a definite behavior or a developmental process” (Karlson and Lüscher, 1959). The search for these semiochemicals is still an elusive goal of chemical ecology and communication studies. Today, it is clear that chemosignals play a significant role in social interaction and communication in various species. It is well known that animals are able to communicate affective states, such as stress, alarm, fear, anxiety, or sexual interest, by manipulating chemosignals produced from their skin (Kiyokawa, 2004; Kiyokawa et al., 2006). In animals, these signals are jointly processed by the vomeronasal organ (VNO) and by the accessory olfactory systems, as well as by the main olfactory system (Chamero et al., 2012; Petrulis, 2013). The wide range of literature addressing the VNO provides little consensus about the presence of the VNO in humans (for a review, see Meredith, 2001). While a structure similar to the VNO of animals was found *in utero* in humans (Knecht et al., 2003), the same structure is not continuously noticeable in adults (Trotier et al., 2000; Trotier, 2011). To date, it is not clear whether a human VNO exists and whether it plays a role in the perception of chemosignals (Frasnelli et al., 2011).

Several areas of the human body—such as feet, mouth, or, particularly, axillary regions—are known to produce odors that act as chemosignals (Pause, 2012). Recently, Gelstein et al. (2011) found that tears convey chemosignals, and even ear wax was proposed to be able to transport chemosensory information (Prokop-Prigge et al., 2014). Nevertheless, human sweat is the most extensively investigated conductor of chemosignals (Porter and Moore, 1981; Lundström et al., 2008; Zhou and Chen, 2008; Zernecke et al., 2010; Albrecht et al., 2011).

Although the human body odor cocktail can contain well over 200 individual components (Zeng et al., 1996), the most intensively studied component is the steroid 4,16-androstadien-3-one (AND). The chemical structure of the molecule AND is very similar to androstenon, a well-known animal pheromone (Melrose et al., 1971). AND's specific cross-sexual characteristics and its impact on human behavior and psychophysiological events, in particular, have drawn much attention in recent research.

The following sections will focus on research addressing the behavioral and psychophysiological effects as well as the neuronal processing of AND, emphasizing its gender-specific and cross-sexual characteristics.

## Behavioral and psychophysiological effects of *AND*

AND is one of the substances known to modulate psychological and physiological states, as well as human behavior in a non-conscious manner (Lundström and Olsson, 2005). It is known that AND is associated with specific cross-sexual characteristics and has recently attracted much attention, particularly regarding its effect on women's psychophysiology (Jacob and McClintock, 2000; Jacob et al., 2001, 2002; Lundström and Olsson, 2005; Wyart et al., 2007). However, there is evidence that AND influences men's psychophysiology and behavior as well (Bensafi et al., 2003, 2004). These gender-specfic effects not only make AND particularly interesting, but also induce an interpretation bias of certain results due to mixed study groups and inextensive study description. Table 1 gives an overview of gender-specific studies and findings.

**Table 1:** **Behavioral and psychophysiological results induced by *AND***.

		**References**
**RESULTS OF STUDIES INVESTIGATING *AND* IN A FEMALE SAMPLE**		
– Reduction of respiratory and cardiac frequency as well as skin conductance and increased body temperature		Grosser et al., 2000; Lundström and Olsson, 2005
– Higher salivary cortisol levels		Wyart et al., 2007
– Increases feeling of being focused		Lundström et al., 2003
		Lundström and Olsson, 2005
– More intense pain perception		Villemure and Bushnell, 2007
– Intensifies intrasexual competition strategies		Parma et al., 2012
– Higher attractiveness ratings of men		Saxton et al., 2008
– Enhances positive mood (feelings of being more relaxed, calm, and free of negative feelings)		Grosser et al., 2000
– Higher sensitivity to AND in fertile women		Lundström and Olsson, 2005
– Induces faster and more pronounced cortical responses		Lundström et al., 2006a
		Lundström et al., 2006b
**RESULTS OF STUDIES INVESTIGATING *AND* IN A MALE SAMPLE**		
– Increases cooperative behavior		Huoviala and Rantala, 2013
**RESULTS OF STUDIES INVESTIGATING *AND* IN MIXED SAMPLES**		
**Women**	**Men**	
– Increases sexual arousal and skin temperature in a sexually arousing context	– Increases sexual arousal and skin temperature in a sexually arousing context	Bensafi et al., 2004
	– Decreases respiration rates in a sexual arousing context	
– Decreases skin temperature and increases skin conductance	– Increases skin temperature and skin conductance	Jacob et al., 2001
– Increased positive feelings	– Increased negative feelings (especially in unpleasant settings)	Jacob and McClintock, 2000; Bensafi et al., 2004
– Increased pain perception		Villemure and Bushnell, 2007

The data from previous behavioral and psychophysiological studies are provided in the following sections categorized according to the gender of the sample.

### Studies investigating *AND* in a female sample

Psychophysiological manifestations related to AND exposure in female subjects have been reported, based on AND detected in salivary cortisol levels (Wyart et al., 2007) and autonomic physiology levels showed a significant reduction in respiratory and cardiac frequency, as well as skin conductance and increased body temperature (Grosser et al., 2000). Beyond that, Lundström and colleagues suggested that AND enhanced women's feeling of being focused (Lundström et al., 2003) and induced an increased attentiveness, even outside the conscious detection of AND (Lundström and Olsson, 2005). In addition, analyses of chemosensory event-related potential (ERP) recordings revealed AND to be processed between 13 and 20 percent faster than odorants similar in hedonic and intensity ratings (Lundström et al., 2006b). With special regard to AND's generally proposed role in reproductive behavior, two studies (Thorne et al., 2002; Saxton et al., 2008) indicate that men's ratings of female attractiveness are modulated by this special chemosignal with higher evaluations of women in the AND condition, while another experiment (Lundström and Olsson, 2005) was not able to confirm this effect.

Saxton et al. (2008), who assessed the effect of AND in a speed-dating-event, argued that the suggested context-dependency of AND may occur only in the presence of a male person (Jacob et al., 2001; Lundström and Olsson, 2005). Furthermore, recent literature suggests that AND strenghtens intrasexual competition strategies in women (Parma et al., 2012).

The role of AND as modulator-chemosignal has also been discussed in the context of serving as a link between hormonal status and this special steroid: While women taking oral contraceptives are more sensitive to environmental odors, fertile women showed a higher sensitivity to chemosignals with reproductive relevance, like androstadienone (Lundström et al., 2006a). With regard to AND's influence on mood, AND was found to enhance a positive mood in women, with feelings of being more relaxed, calm, and free of negative feelings (Grosser et al., 2000; Preti et al., 2003). Another study reported that the setting, the manner, and by whom the experiment was conducted play a role in perception. Lundström and Olsson (2005) emphasized the impact of socioexperimental conditions in women who showed changes in self- reported mood only when experimental interactions were completed by a male experimenter, thus, again, proposing a context-dependent effect for AND.

### Studies investigating *AND* in a male sample

Studies using male samples are rare. The only study investigating the effect of AND in men came out recently, and demonstrated that AND directly affected mens' cooperative behavior by increasing such behavior (Huoviala and Rantala, 2013).

### Studies investigating *AND* in mixed samples

We defined studies with mixed samples as those in which the study design included men and women.

With regard to the activational effects in the sympathetic nervous system, a study by Jacob et al. (2001) was able to confirm the suggested calming effects of AND on women's physiology, as already suggested by a study using the female sample presented in the above section (see Section “Studies Investigating AND in a Female Sample”; Grosser et al., 2000). While AND administration led to raised skin temparature in men and lowered temperature in women, AND increased skin conductance in both sexes, with a significantly higher effect observed in women, indicating that the arousing effect was more prevalent in women than in men (Jacob et al., 2001). Interestingly, the activational effects of AND were dependent on the socioexperimental context, since women's reactions were observed only in sessions administered by a male investigator. Some other findings emphasized the context-dependency of AND in a similar fashion. While AND administered in a neutral context, or in a context with little social interaction, did not influence autonomic nervous system functions, it enabled increased sexual arousal in a sexually arousing context in a sex-independent manner (Bensafi et al., 2004; Hummer and McClintock, 2009). During the same sexually arousing context, respiration rates, especially in men, decreased, while skin temperature in both sexes rose (Bensafi et al., 2004).

Concerning psychological variables, AND was reported to have divergent effects in men and women, as shown for psychophysiological states; while AND administration led to increased negative emotions in men (Jacob and McClintock, 2000), especially in unpleasant settings (Bensafi et al., 2004), no negative effect was evoked in women. Focusing on the context-dependency of AND, positive feelings in women were found to be sustained during a sad time (Bensafi et al., 2004) and were increased in a neutral context (Jacob and McClintock, 2000).

Further, another study that analyzed the effect of AND on mood and pain perception concluded that exposure to this steroid led to an amelioration of mood state only in women (Villemure and Bushnell, 2007). Based on this finding, the authors further hypothesized that women would show lower pain sensation when exposed to AND. However, this assumption was not confirmed, as women, interestingly, showed increased percieved pain (Villemure and Bushnell, 2007).

An effect of the experimenters sex on the baseline response in an AND experiment has recently also been observed in rodents (Sorge et al., 2014). Regarding future research, preliminary findings about the sex-divergent effects of AND on psychophysiological as well as psychological variables should be considered, especially the context-dependency of AND. This context-dependency should implicitly be noted when planning experiments, as well as interpreting results to prevent an interpretation bias.

## Neuronal correlates of *AND*

In the past few years, a great number of neuroimaging studies have provided insight into the neuronal processing of common odorants by the olfactory pathway (for a review, see Lundström et al., 2011). Whereas common odors normally activate the temporal-frontal junction, the so-called piriform cortex, amygdala, insula, and the orbitofrontal cortex, body odors are commonly found to trigger a network located outside the main olfactory system, including the posterior cingulate cortex, the occipital gyrus, the angular gyrus, and the anterior cingulate cortex (for a review, see Lundström and Olsson, 2010). The following section aims to provide the reader with an overview about the neuronal processing of the chemosignal AND.

Neuroimaging studies have illustrated a gender-specific outcome, but another significant finding should be specifically noted—the sexual-orientation effect. As a result of this, the following sections will be segmented into gender-specific neuronal processing and the impact of sexual orientation.

**Figure 1:**
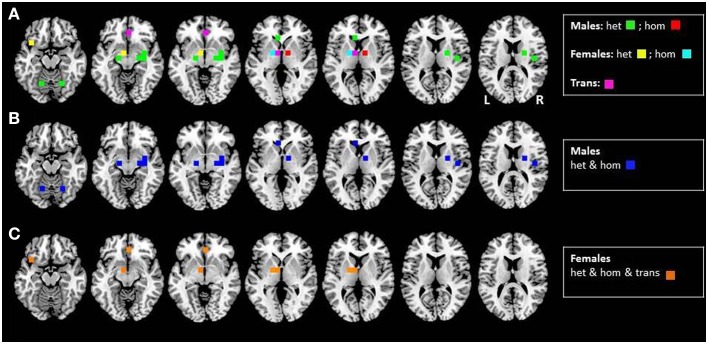
**Axial mean anatomical images overlaid with neural activation induced by AND, resulting from different functional imaging studies (see Table 2).** In order to enhance comparability, we included all positron emission tomography (PET) studies using the same tracer, with the special pheromone-like compound that induced neuronal activation (see Table 2). Since results from certain subject groups were re-utilized as controls throughout these studies, these activations are illustrated once. Voxels were highlighted with a 10 mm sphere. To illustrate the impact of sexual orientation, activations in heterosexual (green) and homosexual (red) men, in heterosexual (yellow) and homosexual (cyan) women, as well as in non-homosexual male-to-female transsexuals, are mapped separately **(A)**. Sex-specific differences in activation patterns are shown in **(B)** (hetero- and homosexual males; blue) and **(C)** (hetero- and homosexual females, as well as male-to-female transsexuals; orange).

To illustrate the findings discussed in the subsequent paragraphs more clearly, we provide the reader with an overview of neural activation patterns induced by AND, classified by gender and sexual orientation (see Figure 1).

**Table 2:** **Overview of studies included in graphic design**.

**References**	**N (total)**	**Healthy adults**		**Modality**	**Stereotactic space**	**Contrast**	**Tracer**
		**Age**	**Subgroups**				
		**(y; range)**	**(sexual orientation)**				
Berglund et al., 2006	36	33 ± 6	12 HeM	PET	TAL	AND > AIR	^15^O-H_2_O
		28 ± 2	12 HoW				
		26 ± 2	12 HeM				
Berglund et al., 2008	36	33 ± 6	12 HeW	PET	TAL	AND > AIR	^15^O-H_2_O
		26 ± 2	12 HeM				
		32 ± 8	12 MFTR's				
Ciumas et al., 2009	26	26 ± 7; 20–36	13 HeW	PET	TAL	AND > AIR	^15^O-H_2_O
		28 ± 6; 21–36	13 HeM				
Hillert et al., 2007	12	26 ± 3; 20–28	12 HeW	PET	TAL	AND > AIR	^15^O-H_2_O
Savic et al., 2001	24	20–28	12 HeW	PET	TAL	AND > AIR	^15^O-H_2_O
		23–28	12 HeM				
Savic et al., 2005	36	26 ± 2	12 HeW	PET	TAL	AND > AIR	^15^O-H_2_O
		28 ± 2	12 HeM				
		33 ± 7	12 HoM				
Savic et al., 2009	12	21-36	12 HeM	PET	TAL	AND > AIR	^15^O-H_2_O

HeW, Heterosexual women; HeM, Heterosexual men; HoW, Homosexual women; HoM, Homosexual men; MFTR's, Non-homosexual male-to-female transsexuals.

### Gender-specific neuronal processing of *AND*

Early positron emisson tomography (PET) studies exploring the neural correlates of AND perception suggested the presence of gender-specific neural activation in which the hypothalamic pathway was significantly activated in heterosexual women, but heterosexual men lacked this hypothalamic activation, and areas of the olfactory cortex were activated instead (Savic et al., 2001, 2005, 2009; Berglund et al., 2006, 2008; Hillert et al., 2007; Ciumas et al., 2009). However, contrary to these PET studies, a recent fMRI study found non-sex-specific hypothalamic activation (Burke et al., 2012). When the authors applied various concentrations of AND, significantly higher hypothalamic activation was demonstrated in women than in men when a higher concentration was used (10 mM). This corresponds with previous studies. However, when participants were exposed to medium concentrations (0.1 mM), men demonstrated a significantly stronger hypothalamic response than the participating women (Burke et al., 2012). These results led the authors to conclude that AND evokes hypothalamic responses in both sexes in a stimulus concentration-dependent manner. However, when comparing these results to the series of studies by Savic and co-workers (Savic et al., 2001, 2005, 2009; Berglund et al., 2006, 2008; Hillert et al., 2007; Ciumas et al., 2009), it should be noted that Burke et al. (2012) used other odor delivery methods, compound concentrations, as well as another imaging technique, all of which together affected our ability to directly compare results.

### Impact of sexual-orientation on neuronal processing of *AND*

The last decade of brain imaging research has revealed that AND stimulation produces significant and localized group effects that are seemingly sexual orientation-dependent. There are several functional neuroimaging studies, which address cortical responses to pheromones that seem to be dependent on sexual orientation; homosexual men and male-to-female transsexuals were found to display the same activation pathway as heterosexual women, i.e., demonstrating hypothalamic responses upon exposure to AND (Savic et al., 2005; Berglund et al., 2008).

In contrast, Berglund and colleagues concluded that homosexual women process AND similar to heterosexual men, namely, by parts of the olfactory cortex (Berglund et al., 2006). Those findings (Savic et al., 2005; Berglund et al., 2006, 2008), are of great importance in order to underline existing evidence of sexual-orientation effects on neural processing in other research areas as well. Recently, behavioral and neuroimaging results from Perry et al. (2013) showed empathy to be related to gender, as well as sexual preference. Further, a study aiming to characterize regional homogeneity and functional connectivity during rest found significant differences between homo- and heterosexual men (Hu et al., 2013). Hence, it would be presumptuous not to consider the ability to use this variable as a modulating factor to achieve homogeneous subject groups.

### AND-EST inconsistency

As noted above, AND is processed in a gender-specific manner; however, thus far, few studies have dealt with the functional aspects beyond neural activity. Most published studies investigating the effect of AND on neural processes also included another potential human pheromone, namely estra-1,3,5(10),16-tetraen-3-ol (EST) (Savic et al., 2001, 2005, 2009; Berglund et al., 2006, 2008; Hillert et al., 2007; Ciumas et al., 2009). EST has, among others, been detected as a natural component of the urine in pregnant women (Thysen et al., 1968). Interestingly, the administration of this estrogen-like steroid causes an effect complementary to AND. Whereas AND application caused a hypothalamic activation in women and the activation of common olfactory areas in men, EST is processed in a diametrically opposite fashion, namely, via the hypothalamic pathway in heterosexual men and parts of the olfactory cortex in heterosexual women (Savic et al., 2001, 2005, 2009; Berglund et al., 2006, 2008; Hillert et al., 2007; Ciumas et al., 2009). This complementary relationship seems to be of great physiological relevance.

Compared to AND, specific psychophysiological measures of EST are largely unknown. However, while the results of Bensafi et al. (2003) revealed no effect of EST on physiological arousal, the same authors found EST to affect physiological arousal in a content-dependent way (Bensafi et al., 2004). Exploring the conscious odor perception of AND and EST in an animal model, a gender-specific effect was obtained by Laska et al. (2006), who detected olfactory sensitivity to AND in female, but not male, spider monkeys, while responses to the highest concentrations of EST were found in males, but not in female, monkeys. These data also highlight the gender-specific processing of these two pheromone-like compounds in non-human animal models. Finally, these results indicate that there is a considerable need for research on the psychophysiological effects of EST.

## Final remarks

As shown, non-conscious application of AND mediates human behavior, psychophysiology, as well as cortical processing, with different responses and activations in men and women. With regard to previous findings, the suggested influence of contextual condition should be considered in any further planning of a trial, as well as in the interpretation and reporting of results. In addition, the discrepancies in results between various studies further emphasize the need for further research in this area, especially in the field of sub- and suprathreshold application of AND to rule out potential concentration-dependent effects. Moreover, the advent of chemosensory imaging using fMRI allows a more stringent and temporally detailed investigation of the neural processing of AND. Finally, as demonstrated by the neuroimaging results presented above, linked to sexual orientation, care should be taken to either include homogeneous subject groups or to carefully control for demographic characteristics. The mediating mechanisms of these sex and sexual preference-specific effects on behavioral, psychophysiological, and neural processing of AND should form the basis of further research.

### Conflict of interest statement

The authors declare that the research was conducted in the absence of any commercial or financial relationships that could be construed as a potential conflict of interest.
